# Assay for Neural Induction in the Chick Embryo

**DOI:** 10.3791/1027

**Published:** 2009-02-13

**Authors:** Delphine Psychoyos, Richard Finnell

**Affiliations:** Institute of Biosciences and Technology, Center for Environmental and Genetic Medicine, Texas A & M University

## Abstract

The chick embryo is a valuable tool in the study of early embryonic development. Its transparency, accessibility and ease of manipulation, make it an ideal tool for studying  the formation and initial patterning of the nervous system. This video demonstrates how to graft organizer tissue into a host, a method by which Hensen s node (the  organizer  in the chick embryo) is grafted to a host  competent  ectoderm. The organizer graft  instructs  overlying  na ve  tissue to adopt a neural fate via neural inducing signals. This mechanism is referred to as neural induction, and constitutes the initial step in the formation of brain and spinal cord in amniotes. This method is essentially used for the characterization of putative neural inducing molecules in chick. This video demonstrates the different steps in the assay for neural induction; First, the donnor embryo is explanted and pinned on a dish. Then, the host embryo is prepared for New culture. The graft is excised and transplanted to the host area pellucida margin. The host is cultured for 18-22 hrs.  The assembly is fixed and processed for further applications (e.g. in situ hybridization). This method was originally devised by Waddington ^1,2^ and Gallera ^3,4^.

**Figure:**
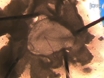


## Protocol

### I. Schematic Overview:


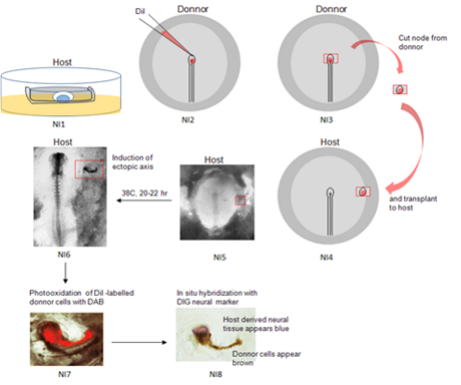


This video demonstrates the different steps in assay for neural induction in chick embryo. First, the host embryo is explanted in New culture [NI1]. Then, the donnor embryo is explanted in saline and Hensen’s node (the “organizer” in chick) is labelled with fluorescent dye DiI [NI2]. Hensen’s node is excised from the donnor embryo [NI3] and transplanted to the host embryo [NI4, NI5]. The host embryo is then cultured for up to 22 hrs, after which period the donnor tissue has induced an ectopic axis in the host area opaca [NI6]. Following culture, the host embryo is fixed and processed for photooxidation in the presence of DAB: By this chemical reaction, donnor derived cells, which appear red under FITC fluorescence prior to photooxidation [NI7], become brown following reaction with DAB [NI8]. The embryo is then processed for in situ hybridization with a neural marker [NI8]; host derived tissue has formed neural tissue in the presence of the donnor graft. This tissue appears blue.

### 1: Preparing the host embryo for New culture

This neural induction assay protocol begins with eggs that have been incubated to Hamburger & Hamilton stage 3^+^ (or HH3^+^). These eggs have been incubated laid on their side in a humid incubator for 13 hours.
Prepare the host embryo for New culture (steps 3.1. to 5.5^9^).
Cover the surface of the host embryo with 200 μl saline. This will facilitate later transfer and positioning of the donnor graft.
Finally, cover the assembly with an inverted Falcon 35 mm culture dish.

### 2: Explanting the donnor embryo in saline

Open the egg by tapping on the shell with forceps and removing pieces of the shell, all around the shell. Remove top of shell and discard.
Remove the thick albumin with forceps, and tilt the yolk sac with coarse forceps so that the embryo faces upwards.
Using fine scissors, cut a square of yolk sac around the embryo. Remove the embryo from the yolk with a spoon, and place in a dish containing PBS.
Using forceps, detach the donnor embryo from area pellucida.
Transfer donnor embryo to a Sylgard covered dish containing PBS.

### 3: Labeling, excising and transplanting the donnor graft

Using an microelectrode puller, pull 50 μl glass microcapillary pipettes. Under the microscope, cut the tip of the micropipette using fine forceps. Place micropipette in a petri dish lined with a ribbon of plasteline.
Prepare the dye solution which will be used to label the graft prior to transplantation: carbocyanine dye DiI (1,1’-dioctadecyl-3,3,3’,3’-tetramethylindocarbocyanine perchlorate): First, prepare a stock of DiI in 1ml absolute ethanol at 0.5%. This stock can be stored at -20C in dark. In a water bath set at 42°C, mix prewarmed 40 μl DiI stock to 360μl 0.3M sucrose. The water bath temperature is necessary in order to prevent precipitation of DiI and formation of insoluble crystals. Briefly spin the solution on a mini spectrafuge (for 5-10 seconds). The DiI solution is now ready to use.
Using insect pins, secure the donnor embryo on the bottom of the Sylgard covered dish.
Back-fill the micropipette with DiI. By applying gentle pressure using an aspirator tube assembly, apply a small bolus of DiI to Hensen’s node. Make sure the entire Hensen’s node is labelled.
Using a microcapillary pipette or a microdissecting knife, cut Hensen’s node (“organizer”, donnor graft).
Mark the ventral side and posterior end of the graft with carmine. This will ensure that the graft is positioned with proper dorso-ventral orientation in step 4.3.
Using a 200μl Gilson pipette, transfer the graft to the host embryo.

### 4: Securing the graft and culturing the host embryo

Remove saline from host embryo, making sure the graft remains positioned in close proximity with host site.
Using a microcapillary pipette or a microdissecting knife, make a small incision (80-100μm) in the area pellucida/area opaca boundary region, at the level of Hensen’s node of host embryo.
Using a microcapillary pipette or a microdissecting knife, position the graft so that ventral side faces towards you and the posterior end is parallel to equivalent region in host embryo.
Remove remaining saline, and process the host embryo for New culture for 81-22 hr (steps 6.1 to 6.6^9^).
The host embryo is then fixed overnight at 4°C (steps 7.1 to 7.7^9^).

### 5: Photooxidise the graft under fluorescence

Under fume hood, prepare a DAB working solution in Tris buffer: Dissolve DAB substrate (3,3’-diaminobenzidine tetrahydrochloride) in Tris buffer (100mM Tris-HCl, pH 7.4) at 500μg/ml; keep solution in dark, on ice.
Wash the embryo twice in PBS for 5 mn each and once in Tris buffer for 5 mn.
Transfer the embryo to a glass cavity slide containing 1 ml Tris buffer.
Replace the Tris buffer solution  with 1.5 ml DAB solution. Dispose of Eppendorf tip in bucket containing a 10% bleach solution in order to decontaminate DAB. Place a coverslip on top of cavity slide.
Under FITC (fluorescein isothiocyanate) fluorescence, focus the objective of the microscope on the graft (donnor cells will appear fluorescent red) and expose to FITC fluorescence until all fluorescent red cells have become brown (this happens as a result of the DiI fluorochrome  being photoconverted to insoluble brown crystals, by exposure to FITC excitation wavelength (488nm) in the presence of DAB. This process takes between 20 mn and 2 hours.
Following completion of the photooxidation reaction, remove the coverslip using fine forceps. Transfer embryo to glass a 20 ml scintillation vial containing PBTw (or PBS with 0.1% Tween-20).
Deactivate DAB from coverslip and glass cavity slide by incubating in 10% bleach solution.

### 6: Process the embryo for in situ hybridization, photography and sectioning

Proceed with in situ hybridization (steps 3.7 to 6.5^9^), incubating the embryo for only DIG labelled probe.
Proceed with photographing (step 8.2 ^9^) and sectioning (steps 8.3 and 8.4^9^).

### Representative Results

In the following example of neural induction assay, the donnor graft is shown induce the expression of the neural marker Tailless from host derived  tissue. In this particular example, we assessed the acquisition of neural inducing ability of embryonic regions which normally do not have “organizer” abilities: the donnor graft originates from an embryo in which the “organizer” region has been surgically removed from the donnor embryo at stage 3^+^. Following ablation, the donnor embryo is allowed to develop in New culture, until the hole has healed and a structure resembling Hensen’s node has formed. This structure is then labelled with DiI, excised from the donnor embryo and grafted to area pellucida/area opaca boundary of a host embryo. Following New culture, a miniature axis is formed adjacent to the host embryo: this miniature axis consists of a rod of red cells (donnor derived) and a head-like structure (host derived): In (A), donnor-derived DiI labelled cells are shown prior to fixation under FITC fluorescence microscopy. These donnor-derived cells have proliferated to form a miniature notochord, which appears as rod of red cells. (B) Following photoconversion of DiI, these donnor-derived cells appear brown. The donnor graft has induced the formation of neural tissue from the host: this tissue consists of precursors for brain, as revealed by their expression for the marker Tailless following in situ hybridization [reprinted from 6].


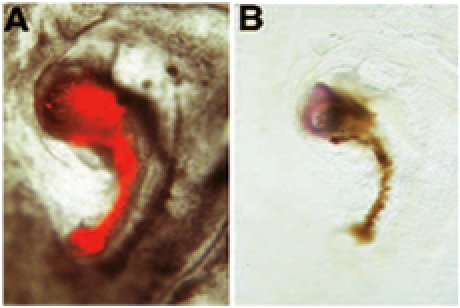


Note: In this procedure, we have used DiI labelled cells in order to differentiate neural inducing donnor tissue (red/brown) from host derived “neural” tissue (blue). Instead of using a chick derived, DiI labelled donnor graft, some authors  also use donnor tissue derived from quail embryos instead of chick [e.g. 6]. This alternative method also allows to differentiate donnor from host tissue. In this case, the graft (donnor tissue) originates from quail eggs instead of chicken eggs. Following step 4.5, the host is processed for whole mount immunohistochemistry with an antibody specific to quail tissue (QCP1) and steps 3.1 to 3.4 and 5.1 to 5.7 are omitted from the protocol. An example of this alternative procedure is shown below: In this case, we used the same experimental procedure as in A,B, but we used quail-derived donnor tissue (as shown by the expression of QCPN antibody [brown]): The regenerated node derived from quail embryo has induced the expression of pan-neural marker Sox2 in the host [reprinted from 6]:


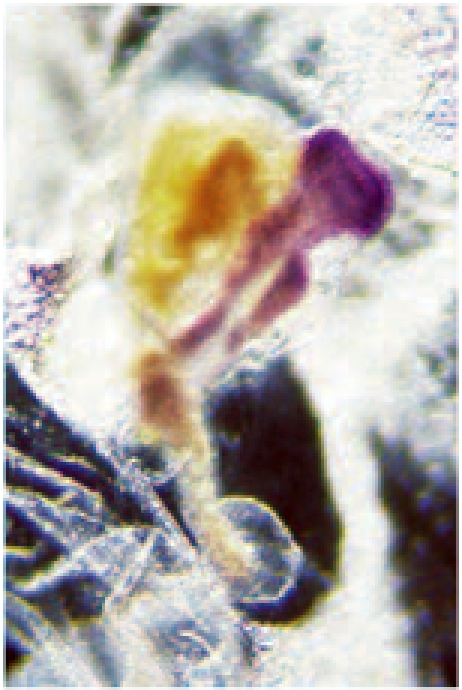


## Discussion

This video demonstrates the different steps in performing an assay for neural induction; This assay is essentially used for the characterization of putative neural inducing molecules in chick, and thus can be used for a wide variety of applications, ranging from embryological micromanipulations ^1-4; 6^ to unraveling new signaling cascades ^7,8^, all aiming to the understanding of the initial step in the formation of the brain and remaining nervous system.

